# Prevalence and Severity of Pain in Cancer Patients in Germany

**DOI:** 10.3389/fpain.2021.703165

**Published:** 2021-09-24

**Authors:** Laura Broemer, Andreas Hinz, Uwe Koch, Anja Mehnert-Theuerkauf

**Affiliations:** ^1^Department of Medical Psychology and Medical Sociology, University of Leipzig, Leipzig, Germany; ^2^Department of Medical Psychology, University of Hamburg, Hamburg, Germany

**Keywords:** pain, cancer, pain assessment, pain prevalence, psycho oncology, quality of life

## Abstract

Pain is a common symptom in cancer patients, restricts daily life activities and reduces survival time. Identification of sociodemographic, medical and psychological correlates of pain among cancer patients in Germany could help identify subgroups most in need of pain management. In this multicenter, epidemiologic cross-sectional study, we assessed pain prevalence and severity, quality of life (QoL) and psychological distress in a sample of 3,745 cancer patients across all tumor entities. In total, 37.9% patients suffered from cancer-related pain and 56.1% suffered from non-specific pain. Younger, female, less educated and unemployed patients reported pain more frequently and more severe pain (*p* < 0.001). Pain was associated with distress, depression, anxiety, QoL, tumor stage (*p* < 0.001), and time since diagnosis (*p* = 0.012). Pain assessment and pain management should be a routine part of cancer treatment and cancer survivorship care plans.

## Introduction

Pain is common among cancer patients and it is one of the most feared symptoms of cancer. Prevalence studies have demonstrated that pain still affects 37–64% of cancer patients (1, 2). Pain is caused by tissue damage produced by the cancer and by treatment-related toxic or traumatic damage to the neural structures (3). Pain can persist after treatment or emerge several months or even years after treatment. Sixty-nine percent of cancer patients who experience pain have reported that pain restricts their daily life activities and 32% reported that they feel so bad they want to die (1). Moreover, studies show that pain in cancer patients remains under-estimated and under-treated (4–8). The negative impact of pain on overall survival has been demonstrated for prostate cancer and is suspected for several other types of cancer (9–11). A large meta-analysis by Quinten et al. (12) found that pain was a prognostic factor for shorter survival, in addition to sociodemographic and clinical factors. Presumably, self-reported pain gives additional information on how far the cancer has progressed and has therefore complementary predictive value to sociodemographic and clinical variables.

Pain prevalence and severity vary considerably, depending on socio-demographic and medical characteristics: Patients with head and neck, lung and breast cancer suffer more often from pain than patients with prostate cancer (13). A female inclination toward greater pain sensitivity has been reported while others found no gender differences (14). Many studies found that older cancer patients report pain less frequently than younger patients (15).

Multiple studies also showed that a cancer diagnosis results in increased levels of distress, depression and anxiety, especially in younger cancer patients and even years after diagnosis (16–19). This psychological burden further increases when patients also suffer from pain and perception of pain is in turn impacted by psychological distress (20–22). Psychological distress and pain affect patients' capability to cope with cancer and burdensome treatment consequences which leads to reduced quality of life (QoL) (23, 24).

Pain has been assessed in several studies in specific cancer populations, such as breast cancer and head and neck cancer patients (23, 25, 26) and in long-term survivors of childhood cancer (27). Mao et al. (28) conducted a large population-based study to assess symptom burden, including pain, in cancer patients. However, to our knowledge, no studies have been carried out during the last decade to investigate pain prevalence and severity and their association with sociodemographic, medical and psychological factors in a large sample across all tumor entities. Assessing pain in a heterogeneous sample of cancer patients with the same instrument will allow to compare pain prevalence and severity between groups of cancer patients with regard to sociodemographic and clinical characteristics. Identification of sociodemographic, medical and psychological correlates of pain among cancer patients could help identify subgroups most in need of pain management. Additionally, pain assessment will be differentiated into cancer-related pain (CRP) and non-specific pain (NSP) in order to explore potential differences. To our knowledge, this has never been investigated so far. The results will potentially contribute to explaining the large variations in findings with regard to pain in cancer patients.

The purpose of this study was (1) to estimate the prevalence and severity of pain in a large representative sample of cancer patients in Germany, (2) to evaluate differences in demographic and medical characteristics between patients with and without pain, and (3) to investigate the associations between pain and distress, anxiety, depression and QoL in this population.

## Materials and Methods

### Study Design

In this multicenter, epidemiologic cross-sectional study, adult cancer patients were consecutively enrolled from acute care hospitals, outpatient cancer care facilities, and cancer rehabilitation clinics in Germany from 2007 to 2011. We used a proportional stratified random sample based on the nationwide incidence of all cancer diagnoses in Germany (29). In this form of stratified random selection, each stratum is represented in the sample in the same proportion as in the population. The University Medical Center Hamburg-Eppendorf coordinated this study. Other participating study centers were the University Medical Centers of Freiburg, Heidelberg, Leipzig, and Würzburg. The ethics committees of all participating centers approved the study protocol. All participants provided written, informed consent. The full study protocol has been published previously (30, 31).

### Patients

Patients with a confirmed diagnosis of a malignant tumor, aged 18–75 years and who were able to speak and read German, were eligible for this study. The exclusion of patients older than 75 years is due to the age limited validity of the Composite International Diagnostic Interview (CIDI) for the Assessment of Comorbid Mental Disorders in Oncology Patients (CIDI-O). Exclusion criteria were the presence of severe physical, cognitive, and/or verbal impairments that interfered with patients' ability to give informed consent.

### Measures

Sociodemographic information was collected through use of a standardized self-report questionnaire; medical information was gathered through medical records [see Mehnert et al. (31) for more detail].

CRP was assessed with a modified version of the Brief Pain Inventory (BPI). Cleeland and Ryan (32) developed the BPI to assess the severity of pain and the impact of pain on daily functions in patients with chronic diseases or conditions such as cancer, osteoarthritis or with acute conditions such as postoperative pain. Radbruch et al. (33) validated the German version of the BPI for patients with CRP and non-cancer related pain. Pain-assessment was two-staged in this study: first, patients were asked “Do you currently have pain that is related to your cancer disease and/or its treatment?” Second, they were asked to rate current pain severity on a 0–10 scale. Only patients, who answered the question on CRP in the affirmative, answered the question on current pain severity. We assigned the score 0 to those participants who answered the question on CRP in the negative.

NSP was assessed with the pain scale from the European Organization for Research and Treatment of Cancer Quality of Life Core Questionnaire (EORTC QLQ-C30). The EORTC QLQ-C30 is a well-validated instrument to measure cancer-related QoL (34). The questionnaire includes, among others, three symptom scales, one of which is the pain scale. The pain scale is composed of two items (“During the past week, have you had pain?” and “During the past week, did pain interfere with your daily activities?”), which are scored on 4-point Likert scales, ranging from 1 (“not at all”) to 4 (“very much”). The pain scale is transformed to a 0–100 range by linear transformation to standardize the raw score. A higher score reflects a higher level of pain. In addition, we dichotomized the first item (“During the past week, have you had pain?” “Not at all” vs. “Any extent”) and analyzed it separately in order to assess the prevalence of NSP (35).

QoL was measured by the global health status/QoL scale from the EORTC QLQ – C30. It is comprised of two items and ranges from 0 to 100, where a higher score reflects a higher QoL.

The German version of the General Anxiety Disorder-scale (GAD-7) was used to assess anxiety (36). The seven items assess the frequency of core symptoms of generalized anxiety disorder within the past 2 weeks. Items are scored on a four-point Likert scale.

The depression module of the German Patient Health Questionnaire (PHQ-9) consists of nine items assessing the frequency of depressive symptoms within the past 2 weeks (37). Items are scored on a four-point Likert scale.

The German version of the NCCN Distress Thermometer is a valid and reliable measure for screening psychological distress in patients with cancer (38). The measure contains a single-item visual analog scale ranging from 0 to 10 to quantify the global level of distress and a problem list. We only analyzed the level of distress in this study.

### Statistical Analysis

The dependent variables were pain prevalence and pain severity as measured with the BPI (CRP) and the pain scale from the EORTC QLQ-C30 (NSP). We report frequencies (*n*) and percentages (%) for categorical characteristics (pain prevalence) and means (M) and standard deviations (SD) for continuous characteristics (pain severity). Chi-square tests were used to test associations of pain prevalence with sociodemographic and clinical characteristics. As a measure of effect size resulting from the Chi-square tests, we present Cramer's V. ANOVAs were used to test associations of pain severity with sociodemographic and clinical characteristics. We present Cohen's f as a measure of effect size resulting from the ANOVAs. Pearson's correlations were calculated to describe associations of pain severity with psychological characteristics. *T*-tests were conducted to test associations of pain prevalence with psychological characteristics. We present the Cohen's d as effect size. For all statistical tests, the result is considered as statistically significant with a 2-sided type-I-error-probability α <0.05. All analyses were performed with SPSS version 25.

## Results

### Participants

**Figure 1:**
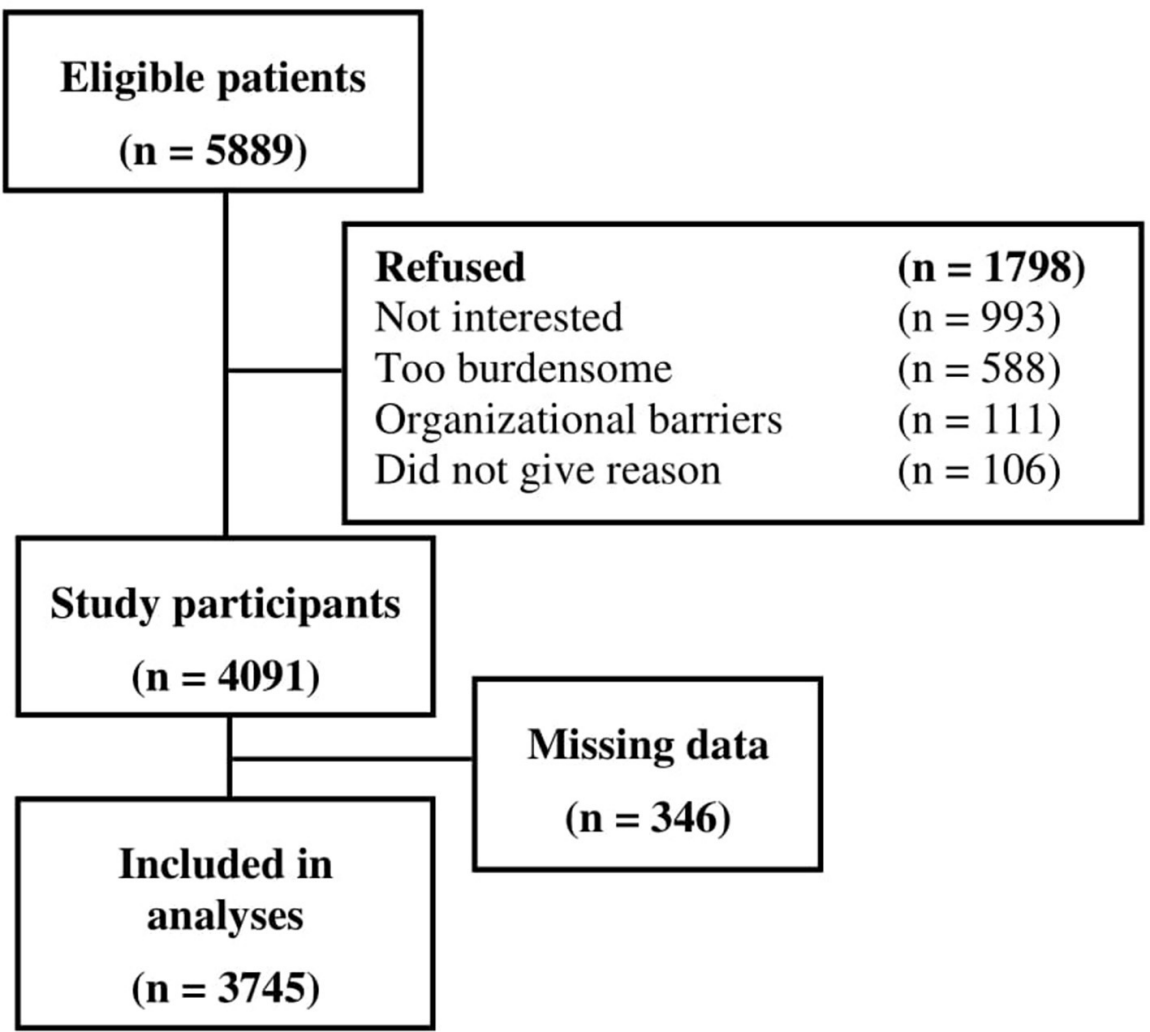
Participant flow including reasons for non-participation.

Out of 5,889 eligible cancer patients, 4,091 participated in the study, leading to a final sample of 3,745 patients with data on pain [Figure 1; for non-responder analyses see (30)]. Demographic and medical characteristics are presented in Table 1. Mean age was 58.1 years (SD = 11.3 years), and mean time since current cancer diagnosis was 13.8 months (SD = 25.7, range 0–318 months).

**Table 1:** Sociodemographic and clinical characteristics with pain as dependent variable.

	**CRP prevalence (BPI)**				**CRP severity (BPI)**			**NSP severity (EORTC QLQ-C30)**		
	***N***	***N* (%)**	***P***	**ES^a^**	**M (SD)**	***p***	**ES^b^**	**M (SD)**	**P**	**ES^b^**
	**Total**	**Yes**								
	3,745	1,420 (37.9)								
**Age^c^**			**<0.001**	**0.11**		**<0.001**	**0.08**		**<0.001**	**0.09**
<45 years	541	230 (45.3)			1.10 (1.92)			35.8 (34.1)		
46–55 years	887	381 (44.5)			1.33 (2.15)			37.0 (34.1)		
56–65 years	1,240	427 (37.0)			1.08 (2.03)			31.3 (33.6)		
>65 years	1,153	359 (31.1)			0.91 (1.92)			30.1 (33.9)		
**Gender**			**<0.001**	**0.08**		**<0.001**	**0.07**		**<0.001**	**0.10**
Female	1,930	807 (41.8)			1.23 (2.14)			36.3 (34.8)		
Male	1,815	613 (33.8)			0.94 (1.88)			29.5 (32.9)		
**School education**			0.082	0.03		**0.028**	**0.03**		**0.001**	**0.06**
≤ 10 years	2,382	928 (39.0)			1.14 (2.09)			34.4 (34.5)		
>10 years	1363	492 (36.1)			0.99 (1.88)			30.5 (33.1)		
**Work situation^c^,^d^**			0.089	0.04		0.099	0.04		**0.001**	**0.07**
Employed	1,505	579 (38.5)			1.05 (1.92)			31.0 (33.2)		
Unemployed	204	91 (44.6)			1.42 (2.23)			40.7 (34.4)		
Retired	1,687	608 (36.0)			1.07 (2.07)			33.3 (34.7)		
Housewife/-husband	180	67 (37.2)			1.01 (2.00)			35.7 (34.6)		
**Tumor entity**			**<0.001**	**0.14**		**<0.001**	**0.11**		**<0.001**	**0.15**
Breast	857	332 (38.7)			1.11 (2.08)			31.7 (33.1)		
Prostate	601	156 (26.0)			0.71 (1.68)			23.9 (30.7)		
Colon/rectum	472	178 (37.7)			0.98 (1.86)			31.9 (34.3)		
Female genital organs	298	137 (46.0)			1.33 (2.17)			42.4 (35.0)		
Hematological cancers	290	102 (35.2)			1.10 (2.10)			33.6 (35.1)		
Lung	285	125 (43.9)			1.37 (2.31)			38.2 (35.1)		
Stomach/esophagus	134	62 (46.3)			1.12 (1.73)			37.1 (34.1)		
Kidney/urinary tract	121	54 (44.6)			1.17 (2.10)			36.4 (31.9)		
Head and neck	119	58 (48.7)			1.55 (2.32)			37.3 (33.5)		
Bladder	80	22 (27.5)			0.90 (2.02)			29.6 (34.2)		
Pancreas	75	34 (45.3)			1.35 (2.11)			37.8 (34.4)		
Malignant melanoma	65	28 (43.1)			1.27 (2.20)			35.6 (35.1)		
Thyroid	24	9 (37.5)			1.50 (2.50)			31.9 (39.5)		
Other	324	123 (38.0)			1.11 (1.94)			35.7 (36.0)		
**UICC stadium^c^**			**<0.001**	**0.13**		**<0.001**	**0.10**		**<0.001**	**0.14**
UICC I	514	164 (31.9)			0.96 (1.92)			30.0 (31.9)		
UICC II	653	219 (33.5)			0.94 (1.87)			28.2 (31.8)		
UICC III	513	176 (34.3)			0.98 (1.96)			29.5 (33.7)		
UICC IV	803	377 (46.9)			1.38 (2.23)			39.1 (35.2)		
**Time since diagnosis^c^**			0.255	0.04		0.334	0.03		**0.002**	**0.07**
0–6 months	1,915	728 (38.0)			1.04 (1.97)			32.3 (33.9)		
7–12 months	581	195 (33.6)			0.97 (1.93)			28.5 (32.1)		
13–24 months	400	151 (37.8)			1.10 (2.01)			34.7 (34.9)		
>24 months	496	189 (38.1)			1.19 (2.15)			35.9 (34.9)		
**Current disease condition^c^**			**<0.001**	**0.11**		**<0.001**	**0.11**		**<0.001**	**0.11**
In remission	1,498	482 (32.2)			0.87 (1.79)			29.6 (31.9)		
Not in remission	1,409	617 (43.8)			1.32 (2.19)			36.8 (35.8)		
**Therapy^c^^,^^d^**			0.244	0.03		0.071	0.04		0.540	0.00
Surgery only	849	303 (35.7)			0.95 (1.88)			31.6 (33.2)		
Radio-/chemotherapy	780	288 (36.9)			1.06 (2.03)			32.5 (33.8)		
Multimodal treatment incl. surgery	1,832	713 (38.9)			1.14 (2.05)			33.2 (34.3)		
**Cancer care setting**			**<0.001**	**0.09**		**0.022**	**0.04**		**<0.001**	**0.07**
Inpatient care	1,585	681 (43.0)			1.19 (2.05)			35.5 (35.7)		
Outpatient care	1,239	435 (35.1)			1.05 (2.06)			30.4 (32.8)		
Inpatient rehabilitation	921	304 (33.0)			0.96 (1.90)			•(32.4)		

a *Effect size for categorical characteristics: **Cramer's V***.

b *Effect size for continuous characteristics: **Cohen's f***.

c *Missing values excluded*.

d *“Other” excluded. Numbers in bold indicate significant values*.

### Pain Prevalence in Association With Sociodemographic and Clinical Variables

In total, 1,420 patients (37.9%) reported CRP (Table 1). Patients who were older than 65 years reported less often CRP than younger patients (Table 1). Men reported less CRP than women. School education and work situation did not correlate with CRP. Patients with prostate and bladder cancer reported less pain than patients with head and neck cancer or patients with cancer of the female genital organs. A higher UICC stage, current disease condition, and cancer care setting were associated with CRP. All effects were small. Time since diagnosis was not associated with CRP prevalence. Two thousand one hundred and twenty-eight (56.1%) patients reported NSP (any extent).

### Pain Severity in Association With Sociodemographic and Clinical Variables

The one-way ANOVA revealed that CRP severity varied significantly for sociodemographic and medical groups (Table 1): Younger patients (<55 years) and female patients showed higher CRP severity than older patients and male patients. Patients with 10 years or less of school education reported higher CRP severity. Tumor entity, UICC stage, current disease condition and cancer care setting were associated with CRP severity: The highest levels of CRP were found in patients with head and neck cancer, in patients with UICC stage IV, in patients with progressing disease and in patients who were recruited from inpatient acute care facilities. The lowest levels of CRP were found in patients with prostate cancer.

With regard to NSP, 847 (22.3%) patients reported mild pain, 839 (22.1%) reported moderate pain, and 442 (11.7%) reported severe pain. Associations of NSP with sociodemographic and medical variables were to a large part the same as the findings with regard to CRP, with a few exceptions: Unemployed patients and patients who were diagnosed with cancer more than 2 years ago reported higher NSP. The highest levels of NSP were found in patients with cancer of the female genital organs.

### Pain, Psychological Burden, and QoL

Patients with CRP showed higher levels of depression and anxiety (small effects, Table 2), had lower QoL, and were more distressed than patients with no CRP. Correlations between pain prevalence and pain severity and depression, anxiety, QoL and distress were significant (Table 3). NSP severity as measured with the pain scale from the EORTC QLQ-C30 showed higher correlations with depression, anxiety, QoL, and distress than CRP severity as measured with the BPI.

**Table 2:** CRP prevalence and psychological burden.

	**CRP yes**	**CRP no**		
	**M (SD)**	**M (SD)**	***p***	***d* (Cohen)**
NSP severity	59.81 (30.01)	16.60 (24.71)	<0.001	1.27
Depression	8.01 (4.90)	5.39 (4.18)	<0.001	0.56
Anxiety	6.32 (4.38)	4.53 (3.76)	<0.001	0.44
Distress	5.39 (2.47)	4.05 (2.50)	<0.001	0.52
QoL	46.10 (21.73)	62.14 (21.52)	<0.001	0.70

**Table 3:** Correlations between pain and psychological variables.

	**CRP prevalence^a^**	**CRP severity**	**NSP severity**	**Depression**	**Anxiety**	**Distress**
CRP severity	0.69					
NSP severity	0.62	0.59				
Depression	0.27	0.30	0.42			
Anxiety	0.21	0.25	0.34	0.61		
Distress	0.25	0.29	0.36	0.50	0.55	
QoL	−0.34	−0.33	−0.53	−0.53	−0.45	−0.54

a *Point biserial correlation; all correlation are statistically significant with a probability of α < 0.001*.

## Discussion

We assessed pain prevalence and severity in a large, representative sample of cancer patients in Germany and found that 37.9% of cancer patients suffer from CRP.

Our results are to a large part in accordance with the extensive review by van den Beuken-van Erverdingen et al. (13) who reported that 37.2–64.1% of cancer patients suffer from pain. Our finding is located on the lower end of this interval. This might be due to the specific wording of the question: we asked specifically for current cancer and cancer treatment related pain, not pain in general. However, when patients were asked for NSP (“During the past week, have you had pain?”), 56.2% answered the question in the affirmative and reported mild, moderate or severe pain. This demonstrates the importance of precise wording, depending on what we want to measure: CRP and pain in cancer patients are not necessarily the same. This partly explains the large variation in findings with regard to pain in cancer patients.

More women than men reported CRP and women also experienced more severe pain than men. Findings with regard to gender differences in cancer pain are inconsistent. While some studies and reviews report no differences between genders (39, 40), others report that women experience more pain than men (41, 42). A review by Fillingim et al. (14) found that the majority of articles investigating gender differences in pain in both cancer and non-cancer setting report either significantly greater pain scores in women or no gender differences. A population based study with 15,386 participants similarly found that men reported less pain than women (43). Reasons for those gender differences put forward by researchers and clinicians are physiologic differences, such as increased serotonergic activity, rendering women more vulnerable to chronic pain. Secondly, women suffer more often from mood disorders, such as anxiety, in consequence of cancer (44, 45) and other comorbidities, which in turn are associated with increased pain. Lastly, gender stereotypes might mediate self-reported pain scores through psychosocial mechanisms (14).

Younger patients reported both more frequently CRP and also higher pain severity than older patients which is consistent with previous studies [e.g., (15)]. Gallaway et al. (46) found that cancer survivors older than 65 reported pain less frequently. Another study similarly found that younger age was associated with higher risk for pain in breast cancer survivors (47). Why older patients are less likely to report pain is not entirely understood: Do they genuinely experience less pain, do they classify pain differently due to previous pain experience or do they just report it less due to stoicism, memory loss, confusion or fear of being a burden (48)? If the latter is true, older cancer patients are in danger of being undertreated for pain. Because of the highly subjective nature of pain, this question is hard to answer, but deserves scientific attention all the more.

Patients with a school education of 10 years or less and unemployed patients reported higher pain severity. The association of pain and (re)employment after cancer has been reported previously: Broemer et al. (49) found higher pain levels in unemployed head and neck cancer patients compared to employed patients. Similarly, Cox-Martin et al. (42) found that only 34% of female cancer patients with pain reported being employed compared to almost 60% without pain. Longitudinal studies are needed to determine a causal direction, since pain may have an impact on employment status or vice versa. Several studies found that household income and educational level were inversely associated with pain prevalence and severity, and with feeling impaired through pain (50–52). This association might be caused by differences in the ability to adequately describe pain and in access to and knowledge of pain medication (53). Due to the temporal order of school education and pain, we conclude that school education has a small impact on pain in cancer patients.

With reference to tumor entities, we demonstrated lower pain prevalence rates and pain severity in prostate cancer (26%) compared to head and neck cancer (48.7%) and cancer of the female genital organs (46.0%). This is in line with van den Beuken-van Erverdingen et al. (13) who also found lower prevalence rates in patients with prostate cancer. Not surprisingly, pain prevalence rates and severity were highest in patients with UICC stage IV who were not in remission and who received hospital treatment. The cancer itself causes pain, and those with more advanced cancer are more likely to experience pain (54). Interestingly, NSP severity in cancer patients decreased during the first year after diagnosis, but increased again after one and two years after diagnosis. This might be because the study sample included only patients who were still receiving treatment and did not include those cancer survivors who were not in need of treatment due to complete recovery and no symptom burden. However, our finding implicates that there is a group of long-term cancer survivors whose pain experience does not alleviate but worsens instead. This highlights the importance of longitudinal after care.

Pain prevalence and pain severity were associated with anxiety, depression, distress and QoL, whereby association with QoL was strongest. A study by Cox-Martin et al. (42) indicated that health-related QoL was significantly lower in cancer patients who experienced pain, particularly for those with uncontrolled pain. Moreover, chronic pain is a risk factor for determining depression (55, 56), and their coexistence further aggravates the severity of both disorders (57). This is partly explained by the common neuroplasticity changes of chronic pain and depression (21). Our finding draws attention to the fact that there is a group of cancer patients not only suffering from pain, but that is also at a higher risk of developing mood disorders and having a lower QoL. Correlations between NSP severity and psychological characteristics were higher than correlations between CRP severity and psychological characteristics. NSP severity was assessed with two items, one of which asked for interference of pain with daily activities. The EORTC QLQ-C30 pain scale therefore not only measures pain severity, but also how much cancer patients feel impaired by this pain. In addition, NSP is by definition a broader construct than CRP and might therefore have more in common with psychological constructs. CRP severity on the other hand was assessed with only one item from the BPI and is limited to a specific kind of pain.

Our study had some limitations. The comparability of our results with regard to CRP are limited since CRP assessment was two-staged in this study: only patients who answered the question on CRP in the affirmative answered the question on CRP severity. Equating CRP severity with zero in those participants who answered the question on CRP prevalence in the negative might underestimate CRP severity: Not everybody negating CRP would necessarily have checked zero on the questions regarding pain severity. It is a reasonable assumption that most of them would have done so, but this approach still leads to a reduced differentiation within the lower range of pain severity and potential underestimation of CRP. On the other hand, excluding 62.1% of the sample (those who answered the question on CRP in the negative) leads to a considerable overestimation of CRP severity and potentially biased result and is not advisable. Therefore, we conclude that including the whole sample by equating no CRP with a pain severity of zero is the more valid method of evaluating the BPI questionnaire. Another limitation of the present study is that CRP and NSP were assessed differently with regard to recall period and item number and range. The assessment of NSP prevalence was not dichotomous as was the assessment of CRP. This reduces the comparability of those two concepts. Additionally, the informative value of our results is restricted to patients younger than 76 years and to patients living in Germany between 2007 and 2011. Results with regard to cancer patients older than 75 years might differ substantially from our results, especially since age seems to impact pain experience.

We assessed pain prevalence and severity in a heterogeneous group of cancer patients and provided insight into which cancer patients are particularly burdened with pain. Future studies need to carefully differentiate between CRP and NSP in cancer patients.

## Data Availability Statement

The raw data supporting the conclusions of this article will be made available by the authors, without undue reservation.

## Ethics Statement

The Ethics Committees of all participating centers approved the study protocol: University Medical Centers of Freiburg, Heidelberg, Leipzig, and Würzburg. The patients/participants provided their written informed consent to participate in this study.

## Author Contributions

LB, AH, and AM-T: conception and design. AM-T: administrative support. AM-T and UK: provision of study materials or patients and collection and assembly of data. LB and AH: statistical analysis. LB: manuscript writing. All authors: final approval of manuscript.

## Funding

This study was funded by a grant from the German Cancer Aid (Grant No: 107465) within the psychosocial oncology funding priority program.

## Conflict of Interest

The authors declare that the research was conducted in the absence of any commercial or financial relationships that could be construed as a potential conflict of interest.

## Publisher's Note

All claims expressed in this article are solely those of the authors and do not necessarily represent those of their affiliated organizations, or those of the publisher, the editors and the reviewers. Any product that may be evaluated in this article, or claim that may be made by its manufacturer, is not guaranteed or endorsed by the publisher.

## References

[1] Breivik H Cherny N Collett B Conno F de Filbet M Foubert AJ. Cancer-related pain: a pan-European survey of prevalence, treatment, and patient attitudes. Ann Onc. (2009) 20:1420–33. 10.1093/annonc/mdp001 19244085

[2] van den Beuken-van Everdingen MHJ de Rijke JM Kessels AG Schouten HC van Kleef M Patijn J. High prevalence of pain in patients with cancer in a large population-based study in The Netherlands. Pain. (2007) 132:3. 10.1016/j.pain.2007.08.022 17916403

[3] Aaronson NK Mattioli V Minton O Weis J Johansen C Dalton SO. Beyond treatment - psychosocial and behavioural issues in cancer survivorship research and practice. EJC Suppl. (2014) 12:54–64. 10.1016/j.ejcsup.2014.03.005 26217166 PMC4250535

[4] Deandrea S Montanari M Moja L Apolone G. Prevalence of undertreatment in cancer pain. A review of published literature. Ann Onc. (2008) 19:1985–91. 10.1093/annonc/mdn419 18632721 PMC2733110

[5] Valeberg BT Rustøen T Bjordal K Hanestad BR Paul S Miaskowski C. Self-reported prevalence, etiology, and characteristics of pain in oncology outpatients. Eur J Pain. (2008) 12:582–90. 10.1016/j.ejpain.2007.09.004 18023377

[6] Greco MT Roberto A Corli O Deandrea S Bandieri E Cavuto S. Quality of cancer pain management: an update of a systematic review of undertreatment of patients with cancer. J Clin Oncol. (2014) 32:4149–54. 10.1200/JCO.2014.56.0383 25403222

[7] Breuer B Chang VT von Roenn JH von Gunten C Neugut AI Kaplan R. How well do medical oncologists manage chronic cancer pain? A national survey. Oncologist. (2015) 20:202–9. 10.1634/theoncologist.2014-0276 25582140 PMC4319627

[8] Sun V Borneman T Piper B Koczywas M Ferrell B. Barriers to pain assessment and management in cancer survivorship. J Cancer Surviv. (2008) 2:65–71. 10.1007/s11764-008-0047-0 18648988 PMC2556887

[9] Armstrong AJ Garrett-Mayer E Ou Yang Y Carducci MA Tannock I de Wit R. Prostate-specific antigen and pain surrogacy analysis in metastatic hormone-refractory prostate cancer. Off J Am Soc Clin Onc. (2007) 25:3965–70 10.1200/JCO.2007.11.4769 17761981

[10] Halabi S Vogelzang NJ Kornblith AB Ou S Kantoff PW Dawson NA. Pain predicts overall survival in men with metastatic castration-refractory prostate cancer. J Clin Onc. (2008) 26:2544–9. 10.1200/JCO.2007.15.0367 18487572

[11] Zylla D Steele G Gupta P. A systematic review of the impact of pain on overall survival in patients with cancer. Support Care Cancer. (2017) 25:1687–98. 10.1007/s00520-017-3614-y 28190159

[12] Quinten C Coens C Mauer M Comte S Sprangers MAG Cleeland C. Baseline quality of life as a prognostic indicator of survival: a meta-analysis of individual patient data from EORTC clinical trials. Lancet Onc. (2009) 10:865–71. 10.1016/S1470-2045(09)70200-1 19695956

[13] van den Beuken-van Everdingen MHJ Hochstenbach LMJ Joosten EAJ Tjan-Heijnen VCG Janssen DJA. Update on prevalence of pain in patients with cancer: systematic review and meta-analysis. J Pain Symp Manag. (2016) 51:1070–90.e9. 10.1016/j.jpainsymman.2015.12.340 27112310

[14] Fillingim RB King CD Ribeiro-Dasilva MC Rahim-Williams B Riley JL 3rd. Sex, gender, and pain: a review of recent clin and experimental findings. J Pain. (2009) 10:447–85. 10.1016/j.jpain.2008.12.001 19411059 PMC2677686

[15] Lundstedt D Gustafsson M Steineck G Malmström P Alsadius D Sundberg A. Risk factors of developing long-lasting breast pain after breast cancer radiotherapy. Int J Rad Onc Biol Phys. (2012) 83:71–8. 10.1016/j.ijrobp.2011.05.065 22079722

[16] Götze H Friedrich M Taubenheim S Dietz A Lordick F Mehnert A. Depression and anxiety in long-term survivors 5 and 10 years after cancer diagnosis. Support Care Cancer Off J Multinat Ass Supp Care Can. (2020) 28:211–20. 10.1007/s00520-019-04805-1 31001695

[17] Inhestern L Beierlein V Bultmann JC Möller B Romer G Koch U. Anxiety and depression in working-age cancer survivors: a register-based study. BMC Can. (2017) 17:347. 10.1186/s12885-017-3347-9 28526007 PMC5438539

[18] Hartung TJ Friedrich M Johansen C Wittchen H Faller H Koch U. The Hospital Anxiety and Depression Scale (HADS) and the 9-item Patient Health Questionnaire (PHQ-9) as screening instruments for depression in patients with cancer. Cancer. (2017) 123:4236–43. 10.1002/cncr.30846 28654189

[19] Hinz A Krauss O Hauss JP Höckel M Kortmann RD Stolzenburg JU. Anxiety and depression in cancer patients compared with the general population. European J Can Care. (2010) 19:361–70. 10.1111/j.1365-2354.2009.01088.x 20030697

[20] Michaelides A Zis P. Depression, anxiety and acute pain: links and management challenges. Postgraduate Med. (2019) 131:438–44. 10.1080/00325481.2019.1663705 31482756

[21] Bamonti PM Moye J Naik AD. Pain is associated with continuing depression in cancer survivors. Psyc Health Med. (2018) 23:1185–92. 10.1080/13548506.2018.1476723 29901408 PMC6354241

[22] Zis P Daskalaki A Bountouni I Sykioti P Varrassi G Paladini A. Depression and chronic pain in the elderly: links and management challenges. Clin Int Aging. (2017) 12. 10.2147/CIA.S113576 PMC5407450 28461745

[23] Cramer JD Johnson JT Nilsen ML. Pain in head and neck cancer survivors: prevalence, predictors, and quality-of-life impact. Otolaryngol Head Neck Surg. (2018) 159:853–8. 10.1177/0194599818783964 29943677

[24] Rodriguez C Ji M Wang H Padhya T McMillan SC. Cancer pain and quality of life. J Hospice Pal Nurs. (2019) 21:507. 10.1097/NJH.0000000000000507 30829932

[25] Hamood R Hamood H Merhasin I Keinan-Boker L. Chronic pain and other symptoms among breast cancer survivors: prevalence, predictors, and effects on quality of life. Breast Can Res Treat. (2018) 167:157–169. 10.1007/s10549-017-4485-0 28861642

[26] Bianchini C Corazzi V Malagò M Bellini T Stomeo F Ciorba A. Pain in head and neck cancer patients: the role of gender. J BUON. (2019) 24:2220–6. 31983086

[27] Schulte FSM Patton M Alberts NM Kunin-Batson A Olson-Bullis BA Forbes C. Pain in long-term survivors of childhood cancer: a systematic review of the current state of knowledge and a call to action from the Children's. Oncol Group Cancer. (2020) 127:35–44. 10.1002/cncr.33289 33112416 PMC7875461

[28] Mao JJ Armstrong K Bowman MA Xie SX Kadakia R Farrar JT. Symptom burden among cancer survivors: impact of age and comorbidity. J Am Board Family Med. (2007) 20:434–43. 10.3122/jabfm.2007.05.060225 17823460

[29] Batzler W Giersiepen K Hentschel S Husmann G Kaatsch P Katalinic A. Krebs in Deutschland 2003–2004: Häufigkeiten und Trends. Berlin: Robert Koch-Institut und Gesellschaft der epidemiologischen Krebsregister in Deutschland e.V. (2008). p. 6.

[30] Mehnert A Brähler E Faller H Härter M Keller M Schulz H. Four-week prevalence of mental disorders in patients with cancer across major tumor entities. J Clin Onc. (2014) 32:3540–6. 10.1200/JCO.2014.56.0086 25287821

[31] Mehnert A Koch U Schulz H Wegscheider K Weis J Faller H. Prevalence of mental disorders, psychosocial distress and need for psychosocial support in cancer patients - study protocol of an epidemiological multi-center study. BMC Psych. (2012) 12:70 10.1186/1471-244X-12-70 PMC3434016 22747671

[32] Cleeland CS Ryan KM. Pain assessment: global use of the Brief Pain Inventory. Ann Acad Med. (1994) 23:129–38. 8080219

[33] Radbruch L Loick G Kiencke P Lindena G Sabatowski R Grond S. Validation of the German version of the Brief Pain Inventory. J Pain Symp Manag. (1999) 18:316–22. 10.1016/S0885-3924(99)00064-0 10517039

[34] Aaronson NK Ahmedzai S Bergman B Bullinger M Cull A Duez NJ. The European organization for research and treatment of cancer QLQ-C30. A quality-of-life instrument for use in international clinical trials in oncology. J Nat Cancer Inst. (1993) 85:5. 8433390 10.1093/jnci/85.5.365

[35] Fayers PM Aaronson NK Bjordal K Groenvold M Curran D Bottomley A. The EORTC QLQ-C30 Scoring Manual. 3rd Ed. Brussels: EORTC Quality of Life Group (2001).

[36] Spitzer RL Kroenke K Williams JBW Löwe B. A brief measure for assessing generalized anxiety disorder: the GAD-7. Arch Int Med. (2006) 166:1092–7. 10.1001/archinte.166.10.1092 16717171

[37] Löwe B Gräfe K Zipfel S Witte S Loerch B Herzog W. Diagnosing ICD-10 depressive episodes: superior criterion validity of the Patient Health Questionnaire. Psychother Psychosom. (2004) 73:386–90. 10.1159/000080393 15479995

[38] Mehnert A Müller D Lehmann C Koch U. Die deutsche Version des NCCN Distress-Thermometers. Empirische Prüfung eines Screening-Instruments zur Erfassung psychosozialer Belastung bei Krebspatienten. Zeitschrift für Psychiatrie, Psychologie und Psychotherapie. (2006) 3:213–23. 10.1024/1661-4747.54.3.213

[39] Miaskowski C. Gender differences in pain, fatigue, and depression in patients with cancer. J Nat Can Inst Monographs. (2004) 32:138–42. 10.1093/jncimonographs/lgh024 15263057

[40] Ahmed Y Popovic M Wan BA Lam M Lam H Ganesh V. Does gender affect self-perceived pain in cancer patients? -A meta-analysis. Ann Pal Med. (2017) 6:S177–84. 10.21037/apm.2017.08.09 29156904

[41] Green CR Hart-Johnson T Loeffler DR. Cancer-related chronic pain: examining quality of life in diverse cancer survivors. Cancer. (2011) 117:1984–2003. 10.1002/cncr.25761 21509777

[42] Cox-Martin E Anderson-Mellies A Borges V Bradley C. Chronic pain, health-related quality of life, and employment in working-age cancer survivors. J Can Surv Res Prac. (2020) 14:179–82. 10.1007/s11764-019-00843-0 31828603 PMC7473420

[43] Nolte S Liegl G Petersen MA Aaronson NK Costantini A Fayers PM. General population normative data for the EORTC QLQ-C30 health-related quality of life questionnaire based on 15,386 persons across 13 European countries, Canada and the Unites States. Eu J Can. (2019) 107:153–63. 10.1016/j.ejca.2018.11.024 30576971

[44] Parás-Bravo P Paz-Zulueta M Boixadera-Planas E Fradejas-Sastre V Palacios-Ceña D Fernández-de-Las-Peñas C. Cancer patients and anxiety: a gender perspective. Int J Environ Res Pub Health. (2020) 17:1302. 10.3390/ijerph17041302 32085538 PMC7175312

[45] Hinz A Herzberg PY Lordick F Weis J Faller H Brähler E. Age and gender differences in anxiety and depression in cancer patients compared with the general population. Eu J Can Care. (2019) 28:e13129. 10.1111/ecc.13129 31290218

[46] Gallaway MS Townsend JS Shelby D Puckett MC. Pain among cancer survivors. Prev Chro Dis. (2020) 17:E54. 10.5888/pcd17.190367 32644924 PMC7367076

[47] Gärtner R Jensen M Nielsen J Ewertz M Kroman N Kehlet H. Prevalence of and factors associated with persistent pain following breast cancer surgery. J Am Med Assoc. (2009) 302:18. 10.1001/jama.2009.1568 19903919

[48] Moye J June A Martin LA Gosian J Herman LI Naik AD. Pain is prevalent and persisting in cancer survivors: differential factors across age groups. J Ger Onc. (2014) 5:190–6. 10.1016/j.jgo.2013.12.006 24495701 PMC4354772

[49] Broemer L Friedrich M Wichmann G Müller J Neumuth T Dietz A. Exploratory study of functional and psychological factors associated with employment status in patients with head and neck cancer. Head Neck. (2021) 43:498–512. 10.1002/hed.26595 33615608

[50] Shangguan X Yu Z Ji L Chen Y Wu H Huang R. Cognition and sociodemographic determinants for effective pain control in patients with cancer pain: a cross-sectional survey in China. Curr Med Sci. (2020) 40:249–56. 10.1007/s11596-020-2167-3 32337686

[51] Herrera-Escobar JP Seshadri AJ Rivero R Toppo A Al Rafai SS Scott JW. Lower education and income predict worse long-term outcomes after injury. J Trauma Acute Care Surg. (2019) 87:104–10. 10.1097/TA.0000000000002329 31033884

[52] Rios R Zautra AJ. Socioeconomic disparities in pain: the role of economic hardship and daily financial worry. Health Psychol Off J Div Health Psych Am Psychol Ass. (2011) 30:58–66. 10.1037/a0022025 21299295 PMC3077089

[53] Im E Guevara E Chee W. The pain experience of Hispanic patients with cancer in the United States. Onc Nurs Forum. (2007) 34:861–8. 10.1188/07.ONF.861-868 17723987 PMC2501107

[54] Freynhagen R Bennett MI. Diagnosis and management of neuropathic pain. BMJ. (2009) 339:b3002. 10.1136/bmj.b3002 19675082

[55] Lee J Franz L Goforth HW. Serotonin syndrome in a chronic-pain patient receiving concurrent methadone, ciprofloxacin, and venlafaxine. Psychosomatics. (2009) 50:321–30. 10.1016/S0033-3182(09)70868-0 19996237

[56] Agüera-Ortiz L Failde I Mico JA Cervilla J López-Ibor JJ. Pain as a symptom of depression: prevalence and clinical correlates in patients attending psychiatric clinics. J Aff Dis. (2011) 130:106–12. 10.1016/j.jad.2010.10.022 21055826

[57] Fishbain DA Cutler R Rosomoff HL Rosomoff RS. Chronic pain-associated depression: antecedent or consequence of chronic pain? A review. Clin J Pain. (1997) 13:116–37. 10.1097/00002508-199706000-00006 9186019

